# Aii810, a Novel Cold-Adapted *N*-Acylhomoserine Lactonase Discovered in a Metagenome, Can Strongly Attenuate *Pseudomonas aeruginosa* Virulence Factors and Biofilm Formation

**DOI:** 10.3389/fmicb.2017.01950

**Published:** 2017-10-10

**Authors:** Xinjiong Fan, Mingjun Liang, Lei Wang, Ruo Chen, He Li, Xiaolong Liu

**Affiliations:** ^1^School of Basic Medical Sciences, Anhui Medical University, Hefei, China; ^2^School of Basic Courses, Guangdong Pharmaceutical University, Guangzhou, China

**Keywords:** biofilm, *N*-acylhomoserine lactonase, quorum sensing, *Pseudomonas aeruginosa*, cold adaptation

## Abstract

The pathogen *Pseudomonas aeruginosa* uses quorum sensing (QS) to control virulence and biofilm formation. Enzymatic disruption of quorum sensing is a promising anti-infection therapeutic strategy that does not rely on antibiotics. Here, a novel gene (*aii810*) encoding an *N*-acylhomoserine lactonase was isolated from the Mao-tofu metagenome for the first time. Aii810 encoded a protein of 269 amino acids and was expressed in *Escherichia coli* BL21 (DE3) in soluble form. It showed the highest activity at 20°C, and it maintained 76.5% of activity at 0°C and more than 50% activity at 0–40°C. The optimal pH was 8.0. It was stable in both neutral and slightly alkaline conditions and at temperatures below 40°C. The enzyme hydrolyzed several ρ-nitrophenyl esters, but its best substrate was ρ-nitrophenyl acetate. Its *k*_cat_ and *K*m values were 347.7 S^-1^ and 205.1 μM, respectively. It efficiently degraded *N*-butyryl-L-homoserine lactone and *N*-(3-oxododecanoyl)-L-homoserine lactone, exceeding hydrolysis rates of 72.3 and 100%, respectively. Moreover, Aii810 strongly attenuated *P. aeruginosa* virulence and biofilm formation. This enzyme with high anti-QS activity was the most cold-adapted *N*-acylhomoserine lactonase reported, which makes it an attractive enzyme for use as a therapeutic agent against *P. aeruginosa* infection.

## Introduction

*Pseudomonas aeruginosa* is a Gram-negative bacterium that thrives in diverse terrestrial and aquatic environments. It is also an opportunistic pathogen of several lower eukaryotes, plants, and animals (Schuster and Greenberg, 2006; Tettmann et al., 2016). In immunocompromised humans, *P. aeruginosa* lead to many severe diseases, such as urinary tract infections, cystic fibrosis (CF), conjunctival erythema, soft tissue infections, abscess, catheter-associated infections, corneal infections, and meningitis (Hentzer and Givskov, 2003). Biofilms are surface-associated communities enclosed within an extracellular matrix (O’Toole, 2003), mostly made up of polysaccharides, lipids, nucleic acids, proteins, and other macromolecules and chemicals (Wei and Ma, 2013). During infection, *P. aeruginosa* can form biofilms that are recalcitrant to antibiotic therapies and immune clearance. Classical antibiotics used to treat *P. aeruginosa* infections are required at concentrations 100- to 1,000-fold higher than the concentrations used to kill their planktonic counterparts (Hentzer et al., 2003). Moreover, a serious side effect of antibiotic therapy is antibiotic resistance (Flamm et al., 2004), because the treatment is based on compounds that aim to kill or inhibit bacterial growth (Hentzer and Givskov, 2003).

Cell–cell communication, also known as quorum sensing (QS), is a procedure of bacterial communication that collectively controls group behaviors and triggers target cells to alter gene expression (Fuqua et al., 1994; Waters and Bassler, 2005). The procedure relies on the production, release, and group-wide detection of small diffusible signal molecules, which are typically *N*-acyl L-homoserine lactones (AHLs) in gram-negative bacteria. AHLs are composed of a homoserine lactone ring linked via a saturated or unsaturated fatty acyl chain, and the chain lengths generally vary from 4 to 18 carbon atoms and in the substitution of a carbonyl at C3-position (Shaw et al., 1997). *P. aeruginosa* possesses two QS systems based on acyl homoserine lactone molecules to orchestrate synchronous production of biofilm formation and virulence factors (de Kievit, 2009). *P. aeruginosa* utilizes this system to regulate many extracellular virulence factors, such as pyocyanin, LasB elastase, LasA protease, and biofilm, which exert its important action in promoting infection (Wagner et al., 2003). AHL synthase LasI synthesizes *N*-(3-oxododecanoyl)-L-homoserine lactone (OdDHL) (Gambello and Iglewski, 1991), which is a pivotal factor in producing several virulence factors and the maturation and differentiation of biofilm (Davies et al., 1998), as well as in the modulation of inflammatory responses (Kravchenko et al., 2008); RhlI AHL synthase synthesizes *N*-butyryl-L-homoserine lactone (BHL) (Ochsner et al., 1994). The observation that AHLs have been found to be one of the QS regulator in *P. aeruginosa* suggests that the enzymatic degradation of QS signal molecules (AHLs), also known as quorum quenching (QQ), has been proved to be most promising and applicable (Kalia and Purohit, 2011), because it is unlikely to pose selective pressure for the development of resistance.

Until now, many AHL-degrading enzymes have been discovered from the metagenome and from several bacteria. On the basis of their catalytic mechanisms, AHL-degrading enzymes could be divided into three families: AHL-lactonases (Dong et al., 2000; Bijtenhoorn et al., 2011b; Fan et al., 2012; Mayer et al., 2015; Morohoshi et al., 2015), AHL-acylases (Lin et al., 2003), and AHL-oxidases (Bijtenhoorn et al., 2011a). AHL-lactonases degrade AHLs by breaking the lactone bond (Dong et al., 2000); AHL-acylases hydrolyze AHLs by degrading the amide linkage and separating the AHL into homoserine lactone and fatty acid (Lin et al., 2003); AHL-oxidases degrade signal molecules by oxidizing the u-1, u-2, and u-3 carbons of the acyl chain of AHL (Chowdhary et al., 2007). Furthermore, some enzymes have shown anti-QS activity against *P. aeruginosa* infection (Bijtenhoorn et al., 2011a; Migiyama et al., 2013; Tang et al., 2015; Valera et al., 2016). Very few reports are available on lactonases with novel characteristics, such as cold adaptation and high hydrolytic activity, because such enzymes are rare. Cold-adapted enzymes exhibit high catalytic efficiency at moderate and low temperatures and are thus versatile biocatalysts in many applications (Rahman et al., 2016).

Mao-tofu, a renowned traditional food in south-central China, is a type of fermented tofu. The primary fermenting agent in is *Mucor* spp., which can degrade the proteins, starches, and lipids of soybean (Zhao and Zheng, 2009). Presumably, several Mao-tofu microbes excrete hydrolytic enzymes. Here, we reported the molecular cloning, identification, and biochemical characterization of one novel cold-adapted *N*-acylhomoserine lactonase derived from the Mao-tofu metagenome. This enzyme showed high activity at moderate and low temperatures. It efficiently degraded BHL and OdDHL, and significantly decreased the production of virulence factors and biofilm in *P. aeruginosa* PAO1.

## Materials and Methods

### Chemicals and Materials

All ρ-nitrophenyl esters, BHL, and OdDHL were purchased from Sigma. T4 DNA ligase, restriction endonuclease, and DNA polymerase were purchased from TaKaRa (Dalian, China) and used according to manufacturer recommendations. E.Z.N.A. Plasmid Mini Kit and E.Z.N.A. Gel Extraction Kit were purchased from OMEGA (Norcross, GA, United States). All other chemicals and reagents were of analytical grade and were purchased from commercial sources, unless otherwise stated.

### Bacterial Strains and Plasmids

*Escherichia coli* DH5α and *E. coli* BL21 (DE3) (Novagen, Madison, WI, United States) were used as the host for molecular cloning. The pUC118 (TaKaRa) and pET-28a (+) (Novagen) were used to construct metagenomic libraries and express the target protein, respectively.

### Metagenomic Library Construction and *N*-Acylhomoserine Lactonase Screening

One metagenomic library was constructed using microbes from Mao-tofu. The total DNA was extracted on the base of the method described before (Zhou et al., 1996; Fan et al., 2012). The purified DNA was incompletely digested with *Eco*RI. DNA fragments within 3.0–10 kb were ligated into *Eco*RI-digested pUC118, and the ligation mixtures were transformed into *E. coli* DH5α. The transformed products were plated onto Luria-Bertani (LB) tributyrin agar plates adding 100 μg/mL ampicillin, 0.1 mM isopropyl-β-D-thiogalactoside (IPTG), 0.1% (v/v) tributyrin, and 0.1 mg/ml rhodamine B. After incubation at 37°C overnight, clones with hydrolysis zones were selected. Then positive clones were further tested for the ability to hydrolyze BHL and OdDHL, which was confirmed by high-performance liquid chromatography analysis. One clone with AHL-lactonase activity was screened. The recombinant plasmid (pUC118 No.1) was sequenced on ABI 377 DNA sequencer.

### Cloning, Expression, and Purification of *N*-Acylhomoserine Lactonase

The putative AHL-lactonase gene *Aii810* was amplified from the pUC118No.1 plasmid by using the primers. *Bam*HI and *Hin*dIII restriction sites were introduced for cloning in the pET-28a (+). The following primers were used: fw (5′-CGCGGATCC ATGTTTTTCAAAGTTAACAACCACG-3′; the *Bam*HI cutting site is underlined) and rv (5′-CCCAAGCTTTTAGCCCTTCAGAAAGTGATGAACG-3′; the *Hin*dIII cutting site is underlined). The procedure was carried out according to the method of Fan et al. (2012). After inducible expression with 0.75 mM IPTG at 30°C for 7 h, cells were harvested and disrupted by sonication. The interest protein was purified by a Ni-NTA His∙Bind column. The purified protein Aii810 were collected and stored at -20°C.

### Determination of Molecular Mass

The molecular mass of the denatured protein was determined by sodium dodecyl sulfate-polyacrylamide gel electrophoresis (SDS-PAGE). A 12% SDS-PAGE was prepared by the previous method (Laemmli, 1970). Proteins were stained with Coomassie brilliant blue G-250.

### Determination of Substrate Specificity

General substrates of esterases/lipases were used to determine substrate specificity of Aii810 as per the study (Fan et al., 2012), with slight modification. The reaction was performed at 20°C for 10 min. The production of ρ-nitrophenol was measured at 405 nm by Labsystems Dragon Wellscan MK3. One unit of enzyme activity was defined as the amount of enzyme that produced 1 μmol of ρ-nitrophenol per minute under the conditions. In each measurement, the effect of non-enzymatic hydrolysis of substrates was considered and subtracted from the value measured when the enzyme was added.

### Effect of Temperature and pH on Activity and Stability of Recombinant Aii810

The optimum temperature was determined by testing esterases/lipases activity at pH 6.8 in temperature ranges of 0–70°C as per the study (Fan et al., 2012), with slight modification. Thermostability was evaluated by pre-incubation of the purified Aii810 for intervals in 50 mM of potassium phosphate buffer (pH 6.8) at 0, 10, 20, 35, 40, and 45°C. Then, the residual enzyme activity was tested. The optimum pH of Aii810 was tested using ρ-nitrophenyl acetate (C2) as a substrate at 20°C. The buffers with final concentration of 50 mM were as follows: citric acid-Na_2_HPO_4_ buffer (pH 5.5–8.0) and barbital sodium-hydrochloric acid buffer (pH 6.8–9.5). Overlapping pH values were used to test that there were no buffer effects on substrate hydrolysis. The pH stability was evaluated after incubation of Aii810 for 12 h at 0°C in the previously discussed solution.

### High-Performance Liquid Chromatography Analysis (HPLC)

Aii810 was measured for degrading of BHL and OdDHL by high-performance liquid chromatography analysis (HPLC). The temperature of this hydrolysis reaction was 20°C. A pH 6.8 potassium phosphate buffer (50 mM) with 5% methanol was used to prepare BHL and OdDHL with the final concentrations of 1 mM. Then, the enzyme samples (31 μg) were added into 1-ml substrates and incubated at 20°C for 30 min. The HPLC method used was described previously (Fan et al., 2012). In each test, the effect of non-enzymatic hydrolysis of BHL and OdDHL was considered and subtracted from the value measured when Aii810 was added.

### Effect of Aii810 on the Formation of Virulence and Biofilm in *P. aeruginosa* PAO1

The method used was described previously, with some modifications (Ishida et al., 1998; Tang et al., 2015; Valera et al., 2016). Briefly, *P. aeruginosa* PAO1, which was grown overnight in LB, was diluted to 10^6^ CFU/ml in a 12-well cell culture plate with different concentrations of sterile Aii810 (0.05, 0.5, or 5 U/ml). Thereafter, one sterile microscope glass coverslip (18 mm × 18 mm × 0.15 mm) was placed in a 12-well cell culture plate, and the plate was incubated for 24 h at 37°C in static conditions. The cell density of the planktonic cells was spectrophotometrically measured at 600 nm. The supernatants were filtered and then used for virulence assays immediately or stored at -20°C. The coverslips were washed mildly with PBS three times to remove non-adhered bacteria and were used for biofilm quantification.

The extracellular LasA protease activity of PAO1 cultivated with different concentrations of Aii810 (0, 0.05, 0.5, or 5 U/ml) was measured as per the study (Ayora and Gotz, 1994). The amount of digested azocasein was spectrophotometrically measured at 415 nm. The pyocyanin production of PAO1 was measured according to the method of Essar et al. (1990), with slight modifications. A 1-ml sample was extracted with 1.5 ml chloroform and then reextracted into 500 μl of 0.2 N HCl. LasB elastase was tested using Elastin Congo Red (Ohman et al., 1980). PAO1 supernatant (1 ml) was mixed with 1 ml Elastin Congo Red (20 mg/ml, pH 7.5), and then incubated for 18 h at 37°C with shaking. Insoluble Elastin Congo Red was removed by centrifugation, and then the supernatant was measured at 495 nm. Quantitative analysis of alginate was measured by the method of Yasuda et al. (1993).

The inhibition of biofilm formed by *P. aeruginosa* PAO1 was tested using crystal violet staining assay (Dong et al., 2001). The coverslips were washed thrice with saline to discard the planktonic cells and air dried for 20 min. Subsequently, the wells were stained with 1% crystal violet solution for 20 min. The stained wells were washed thrice with saline and dried for 20 min, and then the stained crystal violet was dissolved with 95% ethanol. Ten minutes later, the solubilized crystal violet of each coupon was detected by measuring absorbance of crystal violet at 540 nm. The inhibition of biofilm formation against *P. aeruginosa* PAO1 was also characterized by scanning electron microscopy (SEM) as described by Grover et al. (2016). The biofilms samples were coated with platinum and analyzed using SEM (Hitachi SU3500, Krefeld, Germany) using the secondary electron emission mode at 1 kV. Images were collected at 5000× magnifications.

The H_2_O_2_ sensitivity disk assay was carried out as per the study (Hassett et al., 1999), with some modifications. Briefly, *P. aeruginosa* PAO1 pre-incubated in LB broth overnight were centrifuged and resuspended in BHI medium to OD600 = 1. PAO1 was cultivated at 37°C in BHI medium for 6 h adding Aii810 (0, 0.05, 0.5, and 5 U/ml). Then, 100 μl culture was mixed with 3 ml of LB soft agar at 40°C in 0.6% (wt/vol) agar and poured on LB agar plates. Sterile filter paper disks were placed on the soft solid agar, and then spotted with 8 μl of 30% H_2_O_2_. Plates were incubated at 37°C for 8 h, and the diameter of the zone of growth inhibition was measured.

## Results and Discussion

### Screening for *N*-Acylhomoserine Lactonases from a Metagenomic Library

Metagenomic strategy has been developed and successfully applied to exploit novel enzymes (Handelsman, 2004). In this study, the total DNA was extracted from Mao-tofu microbes. A metagenomic library containing approximately 13,200 transformants was constructed to screen AHL-lactonase genes. Restriction digestion analysis of recombinant plasmids from 20 randomly picking clones demonstrated that the average size of insert fragments was 4.0 kb, and sizes ranged from 2.5 to 7.5 kb. Out of about 13,200 transformants, several transformants displayed hydrolysis zones on tributyrin plates, which was based on esterase and lipase activity. One *E. coli* DH5α clone harboring the pUC118No.1 showed AHL-degrading activity.

### Genetic Characterization

**FIGURE 1:**
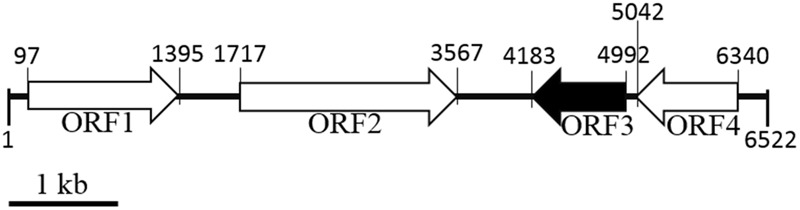
Arrangement of predicted open reading frames on the original genomic clone pUC118No.1. The scale represents a 1-kb length of nucleotides.

**FIGURE 2:**
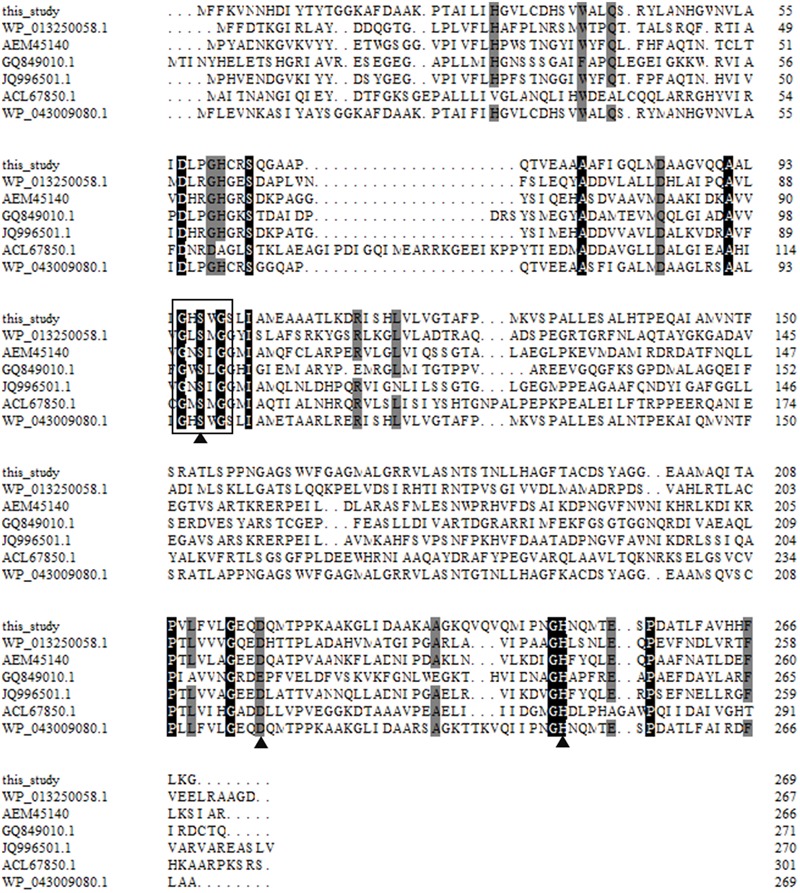
Multiple amino acid sequence alignment of Aii810. Multiple alignment of the partial amino acid sequences containing the conserved motifs of G-X-S-X-G and putative catalytic triad residues of alpha/beta hydrolase family proteins. Except Aii810 (this study), other protein sequences were retrieved from GenBank (http://www.ncbi.nlm.nih.gov). The accession numbers of the aligned sequences are for the following organisms: WP_013250058.1, putative 3-oxoadipate enol-lactonase from *Nitrospira defluvii*; AEM45140, hypothetical protein from uncultured organism; GQ849010.1, alpha/beta hydrolase fold protein from *Ochrobactrum* sp. T63; JQ996501.1, esterase (est816) gene from uncultured bacterium; ACL67850.1, lipolytic enzyme from uncultured bacterium; and WP_043009080.1, alpha/beta hydrolase from *Comamonas testosterone*. The alignment was carried out using the Clustal W method. The open boxes indicate amino acid residues belonging to the putative catalytic triad residues, and triangles denote the active site.

The complete insert DNA sequence of pUC118No.1 was sequenced. Sequence analysis showed that the 6,522-bp nucleic acid fragment consist of four significant open reading frames (ORFs) (**Figure 1**). The third ORF (ORF3, *aii810*) is speculated to encode an enzyme of 269 amino acids, which was related to members of the alpha/beta-hydrolase fold family, with a predicted molecular mass (Mr) of 29.6 kDa. The pI is predicated to be 6.86 (Gasteiger et al., 2005). To measure if ORF3 encodes an *N*-acylhomoserine lactonase, ORF3 was amplified by polymerase chain reaction and ligated into the pET28a. *E. coli* BL21 harboring ORF3-pET28a displayed AHL-degrading activity. Therefore, ORF3 has been named *aii810* (auto-inducer inactivation gene of 810 bp length). This study is the first report on an AHL-degrading gene derived from Mao-tofu microbes, which provided new potential enzyme resources for AHL-degrading genes. The deduced amino acid sequence of Aii810 was used to perform a BLAST program provided by the NCBI and Swiss-Prot databases. There was moderate identity between Aii810 and other hydrolases. The highest identity were the alpha/beta hydrolase fold family protein from *Comamonas testosteroni* (226/267, 85% identity). Multiple sequence alignment of Aii810, putative homologs, and other known AHL-lactonases revealed the typical catalytic triad of active site serine (S97) motif G-X-S-X-G (**Figure 2**), conserved aspartic acid (D218), and histidine (H249) residue motif in the encoded protein (Piper et al., 1993), similar to most lipase/esterases (Fan et al., 2012). This DNA sequence has been submitted to GenBank under accession number (KY783477).

### Heterologous Expression and Purification of Recombinant Aii810

**FIGURE 3:**
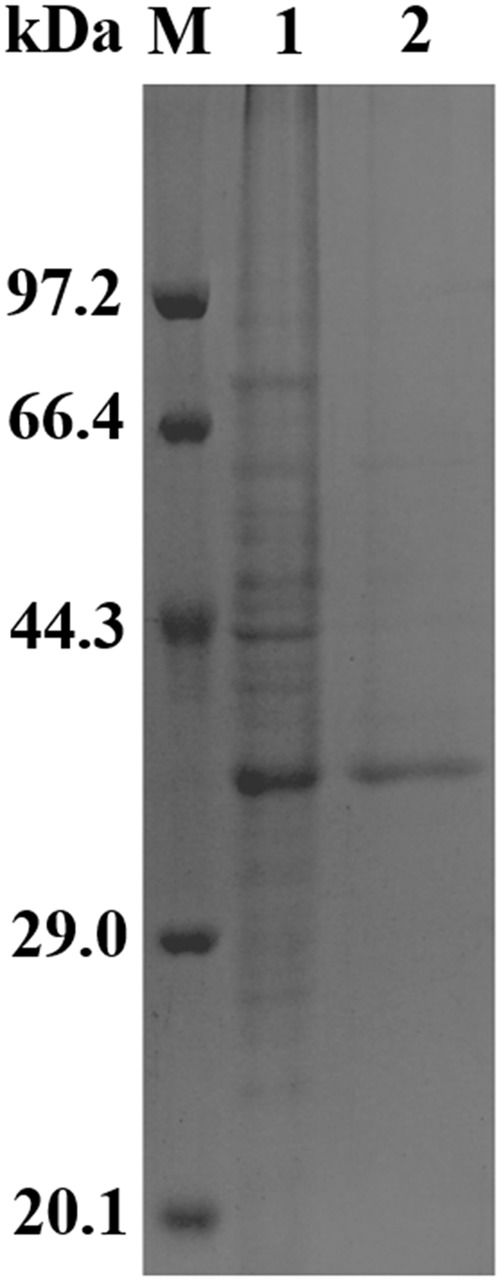
Sodium dodecyl sulfate-polyacrylamide gel electrophoresis analysis of the purified recombinant Aii810. Protein markers (lane M) stained with Coomassie blue, supernatant of *E. coli* BL21 (DE3) cell lysates (lane 1), purified target protein (lane 2). The molecular mass of the enzyme subunit was estimated using the following protein markers as standards: rabbit muscle phosphorylase B (97,200 Da); bovine serum albumin (66,409 Da); ovalbumin (44,287 Da); carbonic anhydrase (29,000 Da); soybean trypsin inhibitor (20,100 Da).

*Aii810* was expressed in *E. coli* BL21 (DE3) and purified. SDS-PAGE analysis demonstrated that the purified enzyme migrates as a single band with a molecular mass a little higher than 35 kDa (**Figure 3**), in accordance with its predicted molecular mass of 35.2 kDa, containing the 269 amino acids and a fusion of 51 amino acids corresponding to polyhistidine tag (His-tag), a unique thrombin cleavage site (Thrombin). The expression quantity was about 0.21 mg/mL (OD_600_ = 3.6). The content of recombinant Aii810 reached up to 50% of the total soluble protein quantified by Quantity One software (Bio-Rad laboratories Inc., Hercules, CA, United States).

### Substrate Specificity and Activity of Aii810

**FIGURE 4:**
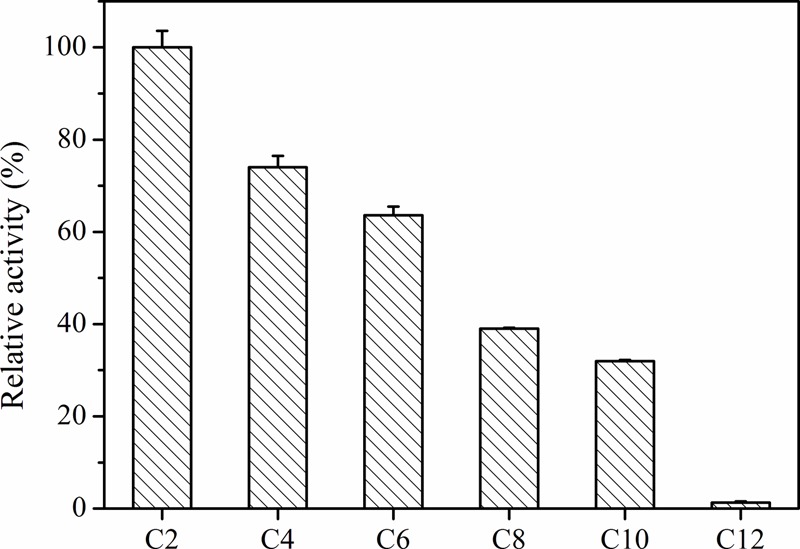
Substrate specificity of Aii810 against various ρ-nitrophenyl esters with acyl chain lengths of C2, C4, C6, C8, C10, and C12. Data points are the average of triplicate measurements, and error bars represent the standard deviation.

Aii810 belongs to the alpha/beta hydrolase. In our research, it revealed a typical catalytic triad of active site serine (S97) motif G-X-S-X-G. For convenience, general substrates of esterases and lipases were chosen to measure substrate specificity of this enzyme. The activity on several ρ-nitrophenyl esters was tested at 20°C and pH 6.5. Aii810 displayed the highest activity toward ρ-nitrophenyl acetate among all tested substrates, and showed lower activity toward with longer acyl esters (**Figure 4**). We calculated the *K*m and *k*_cat_ values by fitting the data to the Michaelis–Menten equation. Using the optimal substrate, the *k*_cat_ and *K*m values of Aii810 were 347.7 S^-1^ and 205.1 μM, respectively. Low *K*m value demonstrated that Aii810 shows positive affinity toward the substrate. High activity is a very attractive property of enzymes for practical applications.

### Effect of pH and Temperature on Recombinant Aii810 Activity and Stability

**FIGURE 5:**
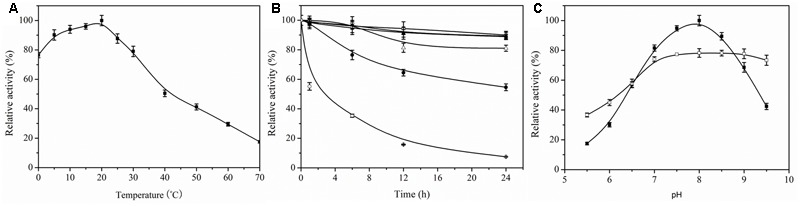
Effect of temperature and pH on activity and stability of recombinant Aii810. Effect of temperature on activity **(A)** and stability **(B)** of recombinant Aii810. The purified enzyme was pre-incubated at 0°C (■), 10°C (□), 20°C (▲), 35°C (△), 40°C (●), and 45°C (○) for different intervals. Effect of pH on activity (■) and stability (□) of recombinant Aii810 **(C)**. The purified enzyme was pre-incubated in different buffers for 12 h at 0°C. Data points are the average of triplicate measurements, and error bars represent the standard deviation.

To measure the optimum temperature of Aii810, the catalytic activity was estimated at different temperatures ranging from 0 to 70°C with ρ-nitrophenyl acetate as a substrate. Aii810 showed the highest activity at 20°C (**Figure 5A**), and retained 76.5% of the maximum activity at 0°C and more than 90% at 5°C. This remarkable activity at cold temperatures indicates that this enzyme could be a cold-adapted protein. Moreover, it maintained more than 50% of its maximal activity at 0–40°C, indicating that Aii810 possessed good adaptability at moderate and low temperatures. Fermentation of Mao-tofu is generally carried out at 20–30°C for 5–6 days (Wu et al., 2015). This demonstrated that Aii810 is endowed with a habitat-specific characteristic (i.e., cold adaptation), because ecological conditions have been found to modulate enzyme characteristics in most cases (Martínez-Martínez et al., 2013).

Thermostability is an important factor to consider in enzyme applications. To analyze thermostability, the proteins were pre-treated at different temperatures, and then the residual activity toward ρ-nitrophenyl acetate was recorded. Incubation at 0–40°C for 12 h reduced the Aii810 activity slightly, but pre-treatment at 45°C for 12 h decreased the activity drastically (**Figure 5B**), suggesting that Aii810 was stable at temperatures below 40°C. Only a few cold-adapted microbial enzymes have shown catalytic activity at such low temperatures. Aii810 has the same optimal temperature as with Lp_2631 from *Lactobacillus plantarum* (Esteban-Torres et al., 2014), slightly lower than other cold-adapted esterases and lipases (Yu et al., 2011; Mander et al., 2012). In the light of cold activity, Aii810 displayed better performance (76.5% at 0°C) than other reported cold-active esterases, such as EstLiu from *Zunongwangia profunda* which showed 75% activity at 0°C (Rahman et al., 2016), est10 from *Psychrobacter pacificensis* which showed 55% activity at 0°C (Wu et al., 2013a), and Est12 from *Psychrobacter celer* 3Pb1 which showed 41% activity at 0°C (Wu et al., 2013b). Another cold-active esterase, EstPc from *Psychrobacter cryohalolentis* K5T, showed 80% of activity at 0°C (Novototskaya-Vlasova et al., 2012). Aii810 showed stronger cold adaptability than any other AHL-degrading enzymes reported previously, such as Est816 (Fan et al., 2012), AiiAAI96 (Cao et al., 2012), and AiiAB546 (Chen et al., 2010), which were most active at 30–60°C. A thermostable *N*-acylhomoserine lactonase AiiT showed maximal activity at higher temperatures ranging from 60 to 80°C (Morohoshi et al., 2015). However, temperature properties of other *N*-acylhomoserine lactonases were not quantified (Dong et al., 2000; Cao et al., 2012; Mayer et al., 2015; Tang et al., 2015; Valera et al., 2016). This enzyme Aii810 was the first cold-adapted *N*-acylhomoserine lactonase, more suitable for application at moderate and low temperatures.

The optimum pH of Aii810 was 8.0. It exhibited poor activity in basic and acidic conditions (**Figure 5C**). Regarding pH stability, it was observed that Aii810 retained more than 70% activity in the pH range 7.0–9.5 but the activity abruptly fell after pH 6.5 (**Figure 5C**). Thus, the pH has an obvious influence on Aii810 activity, which is stable in both neutral and alkaline conditions.

### High-Performance Liquid Chromatography Analysis of AHLs Degradation

**FIGURE 6:**
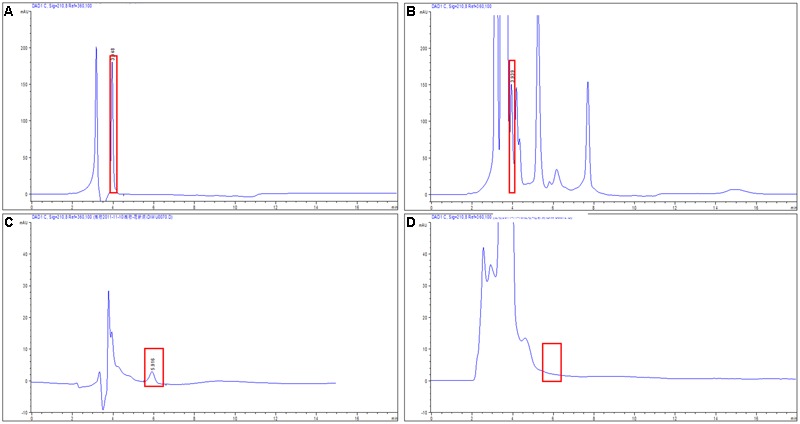
High-performance liquid chromatography analysis (HPLC) analysis of different *N*-acyl L-homoserine lactones hydrolyzed by Aii810. Chromatography of BHL was hydrolyzed by inactive enzyme **(A)** and active enzyme **(B)**, and hydrolysis chromatography of OdDHL by inactive enzyme **(C)** and active enzyme **(D)**. Rectangular box indicates the substrates *N*-acyl L-homoserine lactones before and after hydrolysis.

The AHL-degrading activity of Aii810 was tested using BHL and OdDHL. BHL and OdDHL were chosen as substrates because *P. aeruginosa* uses them as signal molecules to regulate various QS-mediated phenotypes (Pearson et al., 1994). In the natural environment, QS usually happens at 0–40°C. The temperature of this hydrolysis reaction was chose at 20°C. As known to all, AHLs were automatically degraded under alkaline conditions. Considering optimal reaction conditions and the practical application, pH 6.8 potassium phosphate buffer (50 mM) was used to prepare substrates. The degradation efficiency was tested for BHL and OdDHL by HPLC (**Figure 6**). The hydrolysis rates of BHL and OdDHL were 72.3 and 100%, respectively, similar to Est816 and its mutants (Fan et al., 2012; Liu et al., 2016), but distinct from AhlM. AhlM prefered to AHLs with long acyl-chains than short acyl-chains, but it did not degrade BHL (Park et al., 2005). These results suggest that Aii810 has high activity toward these two AHLs and possesses good adaptability at moderate and low temperatures, which may be useful in targeting the QS systems in *P. aeruginosa*, but these findings needs further exploration.

### Effect of Aii810 on the Production of Virulence and Biofilm in *P. aeruginosa* PAO1

**FIGURE 7:**
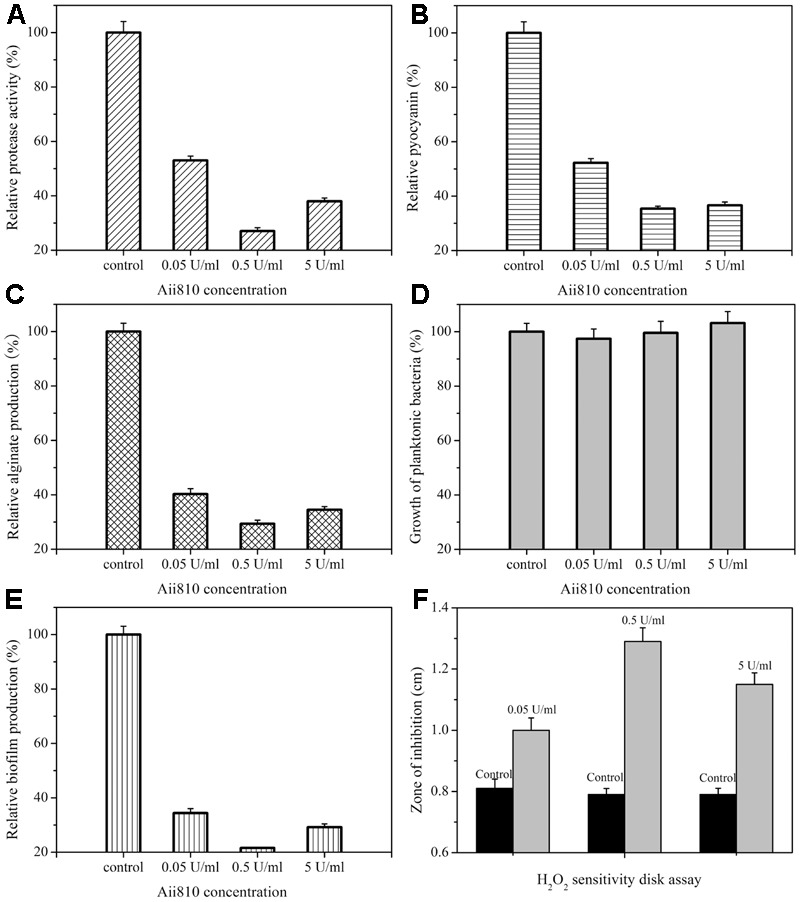
Effect of Aii810 on the production of virulence factors and biofilm, and growth inhibition by H_2_O_2_ of *Pseudomonas aeruginosa* PAO1. Extracellular protease activity **(A)**, pyocyanin production **(B)**, alginate production **(C)**, growth of planktonic bacteria **(D)**, and biofilm production **(E)**. Growth inhibition by H_2_O_2_ of Aii810-treated PAO1 (gray bars) was compared with untreated PAO1 (black bars) **(F)**. All experiments were performed in triplicate. Averages of the triplicate experiments were used to represent each of the three experiments. The data were shown as means ± the standard deviation (SD).

To further explore the anti-QS potential of Aii810, the effects of Aii810 on the production of virulence factors and biofilm in *P. aeruginosa* PAO1 were evaluated *in vitro*. **Figure 7** shows the results. The optimum concentration of Aii810 was 0.5 U/ml. At this concentration, the extracellular protease activity of *P. aeruginosa* PAO1 was reduced by up to 73.4% (**Figure 7A**), outperforming MomL, which decreased 50% extracellular protease activity (Tang et al., 2015). Similarly, adding Aii810 reduced pyocyanin production by *P. aeruginosa* PAO1 by 65.6% (**Figure 7B**). We further demonstrated that the enzyme had a significant effect on alginate production, exceeding an inhibition ratio of about 70.7% (**Figure 7C**).

**FIGURE 8:**
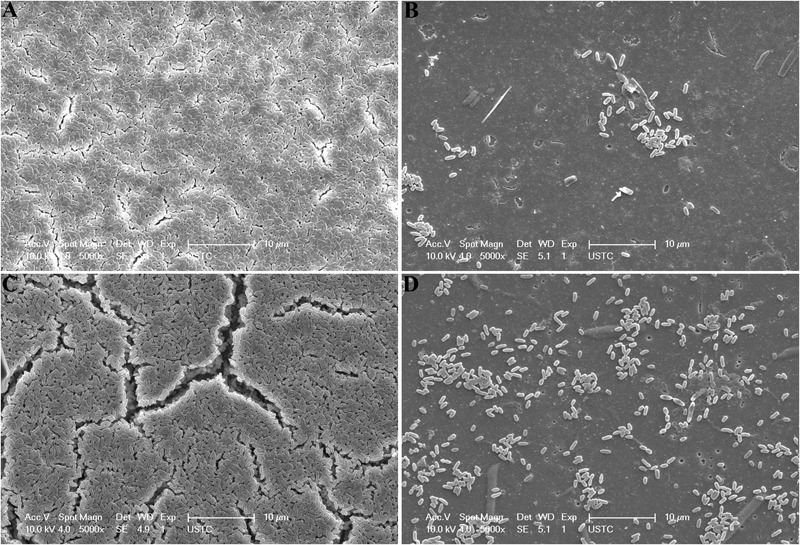
Scanning electron microscopy (SEM) images of biofilm formation by *P. aeruginosa* PAO1 on control glass coverslips without Aii810 at 1 days **(A)** and 2 days **(C)**, and glass coverslips with Aii810 at 1 days **(B)** and 2 days **(D)**. Images were collected at 5000× magnifications.

The inhibition of biofilm formed by *P. aeruginosa* PAO1was also tested. The growth of planktonic bacteria was not affected obviously (**Figure 7D**), whereas biofilm production was significantly reduced by 78.4% (**Figure 7E**), which demonstrated that Aii810 inhibited biofilm without affecting the growth of the bacteria, and unlikely put selective pressure for the development of resistance. The result was better than the antibiofilm activity (70%) of the enzyme from endophytic *Enterobacter aerogenes* VT66 (Rajesh and Rai, 2015). The inhibition of biofilm formation against *P. aeruginosa* PAO1 was also characterized by SEM (**Figure 8**). On the first day, there were regions consisting of a dense network of bacteria on control coverslip (**Figure 8A**). Conversely, in the case of glass coverslip with 0.5 U/ml Aii810, only a few bacteria were observed (**Figure 8B**). On the second day, there was still strong inhibition on biofilm formation (**Figures 8C,D**). Antibiofilm activity of AHL-lactonase from SEM photograph (**Figure 8**) was in accordance with results of biofilm production used to determine inhibition of biomass (**Figure 7E**). Furthermore, electron micrographs of structured colonies showed that the control coverslip was composed of cells surrounded by extracellular matrix (O’Toole, 2003), which was similar to those results observed previously in the biofilms (Branda et al., 2005; Grover et al., 2016).

**FIGURE 9:**
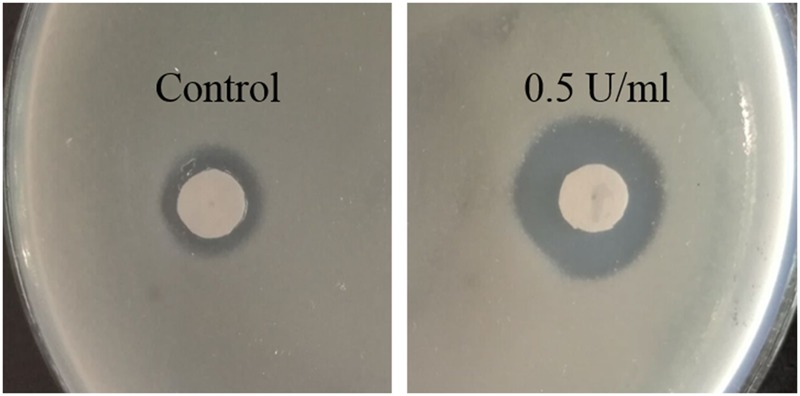
Zone of inhibition by H_2_O_2_ of *P. aeruginosa* PAO1 treated without (control) and with Aii810 at the concentration of 0.5 U/ml.

Quorum sensing signaling in *P. aeruginosa* based on AHLs activates synchronous production of biofilm formation and virulence factors. It also significantly regulated biofilm resistance to H_2_O_2_. Biofilms with less catalase activity would be more resistant to hydrogen peroxide treatment than planktonic bacteria (Hassett et al., 1999). In our result (**Figure 7F**), when exposed to H_2_O_2_ on solid agar, *P. aeruginosa* PAO1 treated with Aii810 were much more susceptible than untreated controls. When the concentration of Aii810 was 0.5 U/ml, the zone of inhibition was the biggest one, as shown in **Figure 9**.

For most reported AHL-lactonases, effects on the production of many virulence factors and biofilm in *P. aeruginosa* have not been quantified (Dong et al., 2000; Park et al., 2003; Mei et al., 2010; Wang et al., 2012; Migiyama et al., 2013; Mayer et al., 2015). Excitingly, we identified a novel AHL-lactonase with cold adaptation and strong inhibition on *P. aeruginosa* virulence and biofilm. These features makes it a potential candidate to disrupt QS in *P. aeruginosa*. Therefore, the inhibition of QS systems might be a more valuable approach than targeting a single particular virulence factor for therapeutic or prophylactic control of infections. The enzyme is of great value for the continued search for a mammalian model.

## Conclusion

Quorum sensing seems to play a key role in the expression of virulence and the interaction with host protection, enzymatic disruption of QS pathways has been suggested to be important components of future anti-pseudomonal therapies (Hentzer and Givskov, 2003). Here, our work is the first report on a novel AHL-lactonase with cold adaptation and high hydrolytic activity from Mao-tofu metagenome. We further demonstrated that the enzyme drastically attenuated *P. aeruginosa* virulence and biofilm. These features makes Aii810 is a potential candidate for use as a therapeutic agent against *P. aeruginosa* infection. Whether this enzyme has an influence on *P. aeruginosa* virulence in tissue culture and animal models; these studies are currently in progress.

## Author Contributions

XF have performed gene cloning, enzyme characterization, and revised the manuscript. ML have performed anti-QS experiments. LW have screened metagenomic library. RC and HL have performed construction of metagenomic library. XL have conceived the study, supervised the experiments, and written the manuscript. All authors have read and approved the manuscript.

## Conflict of Interest Statement

The authors declare that the research was conducted in the absence of any commercial or financial relationships that could be construed as a potential conflict of interest.
